# The Protocatechuate 3,4-Dioxygenase Solubility (PCDS) Tag Enhances the Expression and Solubility of Heterogenous Proteins in *Escherichia coli*

**DOI:** 10.3389/fmicb.2021.779541

**Published:** 2021-11-29

**Authors:** Lei Zou, Sha Li, Nan Li, Shi-Long Ruan, Jing Chen, Jing Wu, Dazhong Yan, Hong-Jun Chao

**Affiliations:** ^1^School of Life Science and Technology, Wuhan Polytechnic University, Wuhan, China; ^2^Daye Public Inspection and Test Centre, Huangshi, China

**Keywords:** fusion tag, heterogenous protein, solubility expression, protocatechuate 3,4-dioxygenase, solubility peptide

## Abstract

*Escherichia coli* has been developed as the most common host for recombinant protein expression. Unfortunately, there are still some proteins that are resistant to high levels of heterologous soluble expression in *E. coli*. Protein and peptide fusion tags are one of the most important methods for increasing target protein expression and seem to influence the expression efficiency and solubility as well. In this study, we identify a short 15-residue enhancing solubility peptide, the PCDS (protocatechuate 3,4-dioxygenase solubility) tag, which enhances heterologous protein expression in *E. coli*. This PCDS tag is a 45-bp long sequence encoding a peptide tag involved in the soluble expression of protocatechuate 3,4-dioxygenase, encoded by the *pcaHG98* genes of *Pseudomonas putida* NCIMB 9866. The 45-bp sequence was also beneficial for *pcaHG98* gene amplification. This tag was shown to be necessary for the heterologous soluble expression of PcaHG98 in *E. coli*. Purified His_6_-PcaHG98e04-PCDS exhibited an activity of 205.63±14.23U/mg against protocatechuate as a substrate, and this activity was not affected by a PCDS tag. This PCDS tag has been fused to the mammalian yellow fluorescent protein (YFP) to construct YFP-PCDS without its termination codons and YFPt-PCDS with. The total protein expressions of YFP-PCDS and YFPt-PCDS were significantly amplified up to 1.6-fold and 2-fold, respectively, compared to YFP alone. Accordingly, His_6_-YFP-PCDS and His_6_-YFPt-PCDS had 1.6-fold and 3-fold higher soluble protein yields, respectively, than His_6_-YFP expressed under the same conditions. His_6_-YFP, His_6_-YFP-PCDS, and His_6_-YFPt-PCDS also showed consistent fluorescence emission spectra, with a peak at 530nm over a scanning range from 400 to 700nm. These results indicated that the use of the PCDS tag is an effective way to improve heterologous protein expression in *E. coli*.

## Introduction

Since the mammalian hormone somatostatin was first used to realize heterologous expression in *Escherichia coli* (Itakura et al., 1977), the expression of recombinant protein has developed rapidly for various applications, including industrial enzyme production (Headon and Walsh, 1994), biopharmaceuticals (Jozala et al., 2016), and vaccine production (Christodoulides et al., 2001). The production of recombinant proteins has been implemented in many different prokaryotic and eukaryotic host systems, including *E. coli*, yeast, filamentous fungi, insect cells, *Arabidopsis*, and even in mammalian cell culture lines (Demain and Vaishnav, 2009). Among these systems, *E. coli* is often preferred due to its fast growth, high cell density cultures, rich complex media, and easy transformation (Rosano and Ceccarelli, 2014). In order to improve the heterologous protein expression of *E. coli* systems, a number of protein tags have been developed, including the maltose-binding protein (MBP; Kapust and Waugh, 1999), glutathione S-transferase (GST; Smith and Johnson, 1988), small ubiquitin-related modifier (SUMO; Marblestone et al., 2006), N-utilization substance protein A (NusA; Davis et al., 1999), thioredoxin (Trx; LaVallie et al., 1993), and ubiquitin (Bachmair et al., 1986; Varshavsky, 2005). In addition, there are some short peptide tags for *E. coli* expression systems wherein the amino acid sequences are generally 15 residues or less (Ki and Pack, 2020), including the Arg-tag (Sassenfeld and Brewer, 1984), FLAG-tag (Hopp et al., 1988), His-tag (Hochuli et al., 1987), c-myc-tag (Ferrando et al., 2001), S-tag (Karpeisky et al., 1994), Strep-tags (Schmidt and Skerra, 1993), and Fh8 and H tags (Costa et al., 2013). These peptide tags can improve the solubility of a target protein by regulating the process of protein transcription and translation (Ki and Pack, 2020). However, a specific fusion tag is not suitable for the protein expression of all proteins (Zhao et al., 2019), and the function of an expressed protein may be negatively affected by the specific fusion tag used for expression (Arnau et al., 2006). Therefore, the identification of additional fusion protein and peptide tags is conducive to finding more beneficial common features for protein heterogeneous expression.

The chemical 2,4-xylenol, known to be harmful to human health, is listed as a toxic pollutant by the U.S. Environmental Protection Agency due to its damage to the environment. The two compounds 2,4-xylenol and *p*-cresol can be catabolized together and have the same enzymes to catalyze the oxidation of *para*-methyl groups in *Pseudomonas putida* NCIMB 9866 (Chen et al., 2014). Moreover, the metabolism of 2,4-xylenol and *p*-cresol is through the protocatechuate (3,4-dihydroxybenzoate, PCA) pathway (Elmorsi and Hopper, 1977; Chen et al., 2014; Chao et al., 2016). Protocatechuate is further metabolized through the *ortho* ring cleavage pathway. *pcaHG* genes encoding protocatechuate-3,4-dioxygenase are located on the *pch* gene cluster in *P. putida* NCIMB 9866 (Chen et al., 2014). In this study, we found a 45-bp-coded peptide tag involved in the soluble expression of protocatechuate 3,4-dioxygenase encoded by the *pcaHG* genes of *P. putida* NCIMB 9866 in *E. coli*. These *pcaHG* genes were cloned and expressed *in vitro*, and a 45-bp sequence termed as the PCDS tag (protocatechuate 3,4-dioxygenase solubility tag) required for its soluble expression is characterized. PcaHG98 appears as an inclusion body without the PCDS tag in *E. coli*. Moreover, this tag could also significantly promote the heterogenous soluble expression of yellow fluorescent protein (YFP), which has relatively low expression levels in *E. coli*.

## Materials and Methods

### Strains, Plasmids, Culture Media, Primers, and Chemicals

The strains used in this study are listed in Table 1. *Pseudomonas putida* NCIMB 9866 was grown at 30°C in minimal medium (MM; Chapman and Hopper, 1968) on different compound carbon sources. *Escherichia coli* was grown in lysogeny broth (LB) on a rotary shaker (200rpm) at 37°C under aerobic conditions. The culture medium is usually supplemented with antibiotics at a final concentration of 50μgml^−1^ for kanamycin sulfate (Kan) and 100μgml^−1^ for ampicillin sodium (Amp), as necessary. The chemicals used in the experiments were purchased from Aladdin Reagents (Shanghai, China) or Sigma-Aldrich (St. Louis, MO, United States). *Taq* DNA polymerase and *Pfu* DNA polymerase were obtained from TaKaRa Bio (Dalian, China). *TransEco FastPfu* DNA Polymerase was purchased from TransGen Biotech (Beijing, China).

**Table 1:** Strains or plasmids used in this study.

Strain or plasmid	Characteristic(s) ^a^ or purpose	Reference or source
*Pseudomonas putida* NCIMB		
9,866	2,4-xylenol and *p*-cresol utilizer, wild-type strain, Amp^R^, Kan^S^, Tc^S^	Chapman and Hopper, 1968
*Escherichia coli*		
*Trans*1-T1 Phage Resistant	F^−^ φ80(*lacZ*) ΔM15 Δ*lac*X74 *hsdR* (r_k_^−^m_k_^+^) Δ*recA*1398 *endA1 tonA*	TransGen Biotech
BL21(DE3)	F^−^ *ompT hsdS* (r_B_^−^m_B_^+^) *gal dcm* (DE3)	Novagen
Plasmid		
pET-28a (+)	Expression vector, Kan^R^	Novagen
pET-28a-*pcaHG98e01*	Expression vector for *pcaHG*, PCR amplified with primers pcahg9803 and pcahg9804 from strain NCIMB 9866 genome, inserted into the *Nde*I-*Hin*d III restriction sites in pET-28a (+)	This study
pET-28a-*pcaHG98e02*	Expression vector for *pcaHG*, PCR amplified with primers pcahg9801 and pcahg9802 from strain NCIMB 9866 genome, inserted into the *Nde*I-*Hin*d III restriction sites of pET-28a (+)	This study
pET-28a-*pcaHG98e03*	Expression vector for *pcaHG*, PCR amplified with primers pcahg9802 and pcahg9803 from strain NCIMB 9866 genome, inserted into the *Nde*I-*Hin*d III restriction sites of pET-28a (+)	This study
pET-28a-*pcaHG98e04*	Expression vector for *pcaHG*, PCR amplified with primers pcahg9801 and pcahg9804 from strain NCIMB 9866 genome, inserted into the *Nde*I-*Hin*d III restriction sites of pET-28a (+)	This study
pET28a-YFP	Expression vector for the cDNA sequence of YFP synthesized according to the sequences from pcDNA3YFP, inserted into the *Nde*I-*Hin*d III restriction sites of pET-28a (+)	This study
pET28a-YFPt-PCDS	Expression vector for the cDNA sequence of YFP synthesized according to the sequences of pcDNA3YFP and PCDS tag with a stop codon, inserted into the *Nde*I-*Hin*d III restriction sites of pET-28a (+)	This study
pET28a-YFP-PCDS	Expression vector for the cDNA sequence of YFP synthesized according to the sequences of pcDNA3YFP and PCDS tag without a stop codon, inserted into the *Nde*I-*Hin*d III restriction sites of pET-28a (+)	This study
pEGFP-N1	Vector encoding a red-shifted variant of wild-type GFP that has fluorescence activity in mammalian cells	Clontech
pET-28a-eGFP	Expression vector for the eGFP gene of pEGFP-N1, inserted into the *Nde*I-*Hin*d III restriction sites of pET-28a (+)	This study
pET-28a-eGFP-PCDS	Expression vector for the eGFP gene of pEGFP-N1 and PCDS tag, inserted into the *Nde*I-*Hin*d III restriction sites of pET-28a (+)	This study

a Amp^R^ resistant to ampicillin; Kan^S^, Tc^S^, sensitive to kanamycin and tetracycline, respectively.

### Gene Cloning and Expression, Protein Purification

**Figure 1:**
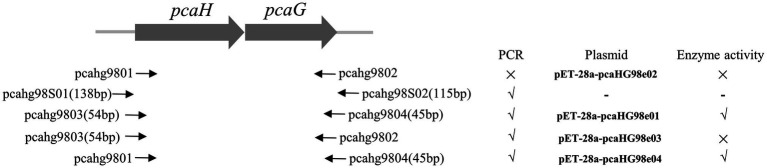
Primers, PCR reactions, plasmids, and enzyme activity information used in this study. Primers are shown with a solid arrow. PCR without product (×) and with product (√). Enzyme without activity (×) and with activity (√).

Plasmid preparation and DNA manipulation were carried out as described previously (Green and Sambrook, 2012). The primers used in this study are listed in Table 2 and are shown in Figure 1. All the targeted genes were amplified by PCR using *Taq* DNA polymerase or *TransEco FastPfu* DNA Polymerase (Transgen, Beijing, China), according to its manufacturer’s recommendation. The *pcaHG98* gene was amplified with PCR with primers pcahg9803 and pcahg9804 from the extracted genomic DNA of strain NCIMB 9866. The PCR reaction volume usually uses 50μl system and consists of 5–30ng Plasmid DNA or 100ng Genomic DNA, 1x *TransEco FastPfu* Buffer, 250μM dNTP, 0.4μM of each primer, 2.5U of *TransEco FastPfu* DNA Polymerase or *Taq* DNA polymerase and ddH_2_O up to 50μl. PCR amplification was performed with *TransEco FastPfu* DNA polymerase as follows: denaturation at 95°C for 5min, 30cycles of 95°C for 1min, 58°C for 1min, and 72°C for 1min (1kb/min for *Taq*, 2kb/min for *FastPfu*), and a final extension cycle at 72°C for 5min. The PCR fragments were digested with *Nde*I/*Hin*dIII (Thermo Fisher Scientific, Shanghai, China) before being cloned into pET-28a (+) with T4 DNA ligase (TaKaRa, Dalian, China) to produce pET-28a-*pcaHG98e01*. The *pcaHG98* gene was amplified using primers pcahg9801 and pcahg9802 from plasmid pET-28a-*pcaHG98e01* and cloned into *Nde*I/*Hin*dIII sites of pET-28a (+) with T4 DNA ligase to produce pET-28a-*pcaHG98e02*. The *pcaHG98* gene was amplified using primers pcahg9802 and pcahg9803 from plasmid pET-28a-*pcaHG98e01* and cloned into *Nde*I/*Hin*dIII sites of pET-28a (+) with T4 DNA ligase to produce pET-28a-*pcaHG98e03*. The *pcaHG98* gene was amplified using primers pcahg9801 and pcahg9804 from plasmid pET-28a-*pcaHG98e01* and cloned into *Nde*I/*Hin*dIII sites of pET-28a (+) with T4 DNA ligase to produce pET-28a-*pcaHG98e04*.

**Table 2:** Primers used in this study.

Published Primer	Sequence (5'-3')	Underlined comment
pcahg98S01	CGCGAGTCGAATGAGGTTGG	
pcahg98S02	CAGCCATTGACCTAGCAGGTC	
pcahg9801	GGGAATTCCATATGTCCGACGCTCAAGAACGTCG	NdeI
pcahg9802	CCCAAGCTTGAAGTCAAAAAAGACTGTCTCG	HindIII
pcahg9803	GGGAATTCCATATGCTCGGCTCCGTTCGATTCAACG	NdeI
pcahg9804	CCCAAGCTTGGGAGACGCGTTTCGTACTCGTG	HindIII
pYFP01	CCGCGCGGCAGCCATATGGTGAGCAAGGGCGAGGAG	15bp overlap for fusion
pYFP02	TCGAGTGCGGCCGCATTACTTGTACAGCTCGTCCATGCC	15bp overlap for fusion
pYFP03	CGTACTCGTGCCGGAGCAGCTGCCCCGGCGATGTCTTACTTGTACAGCTCGTCCATGCC	15bp overlap for fusion and partial PCDS tag
pYFP04	TCCGGCACGAGTACGAAACGCGTCTTAATGCGGCCGCACTCGAGCACCACC	15bp overlap for fusion and partial PCDS tag
pYFP05	CGTACTCGTGCCGGAGCAGCTGCCCCGGCGATGTCCTTGTACAGCTCGTCCATGCCG	15bp overlap for fusion and partial PCDS tag
pegfp03	CCGCGCGGCAGCCATATGGTGAGCAAGGGCGAGGAGC	15bp overlap for fusion
pegfp04	GACGGAGCTCGAATTCTTACTTGTACAGCTCGTCCATGCC	15bp overlap for fusion
pET28a-pcas03	CAGCTGCTCCGGCACGAGTACGAAACGCGTCTCAAGCTTGCGGCCGCACTCG	15bp overlap for fusion and partial PCDS tag
pET28a-pcas04	GTGCCGGAGCAGCTGCCCCGGCGATGTCTCACTTGTACAGCTCGTCCATGCC	15bp overlap for fusion and partial PCDS tag

The nucleotide sequence for YFP was synthesized according to the sequences of pcDNA3YFP, which was a gift from Doug Golenbock (Addgene plasmid # 13033; http://n2t.net/addgene:13033; RRID: Addgene_13,033). The YFP gene was amplified using primers pYFP01 and pYFP02 from this synthesized YFP gene (Sangon Biotech, Shanghai, China) and fused to the *Nde*I/*Hin*dIII restriction sites of pET-28a (+) with the ClonExpress II One Step Cloning Kit (Vazyme, Nanjing, China) to produce pET-28a-YFP. The YFP gene was amplified using primers pYFP03 and pYFP04 from pET-28a-YFP and fused to the *Nde*I/*Hin*dIII restriction sites of pET-28a (+) to produce pET-28a-YFPt-PCDS. The YFP gene was amplified by PCR using primers pYFP04 and pYFP05 and fused to the *Nde*I/*Hin*dIII restriction sites of pET-28a (+) to produce pET-28a-YFP-PCDS. PCR amplification conditions are the same as *pcaHG98* gene described above.

The eGFP gene was amplified using primers pegfp03 and pegfp04 from the mammalian expression vector pEGFP-N1 and fused to the *Nde*I/*EcoR*I restriction sites of pET-28a (+) with the ClonExpress II One Step Cloning Kit to produce pET-28a-eGFP. The primers pET28a-pcas03 and pET28a-pcas04 containing PCDS tag were used to amplify linearized recombinant vector from pET-28a-eGFP, which was then fused by ClonExpress II One Step Cloning Kit to form cyclized vector pET-28a-eGFP-PCDS.

The nucleotide sequence of the resulting plasmid was confirmed by Sangon Biotech (Shanghai, China).

Heterologous expression of the cloned *pcaHG* gene was accomplished by introducing the constructed plasmid into *E. coli* BL21 (DE3; Novagen, Madison, WI). The transformed cells were grown at 37°C to an OD_600_ of 0.4 in 100ml LB supplemented with 50gml^−1^ of kanamycin in 500ml shake flask; then, the protein expression was induced with 0.1mM of isopropyl-β-D-thiogalactopyranoside (IPTG) for approximately 5h at 30°C, resulting in OD_600_ of 2. His_6_-PcaHG98e04-PCDS was purified using Ni^2+^-nitrilotriacetic acid agarose chromatography (Novagen) and eluted with 200mM of imidazole. Purified target His_6_-PcaHG98e04-PCDS was further dialyzed away from imidazole with a Spectra/Por CE dialysis membrane with a molecular weight cut-off of 10,000Da (Spectrum Laboratories Inc., Shanghai, China) at 4°C for 48h against phosphate buffer (PB) before being further preserved in glycerol at −80°C.

The expression and purification of YFP and eGFP are performed according to that of His_6_-PcaHG98e04-PCDS. The total protein amounts of *E. coli* BL21 (DE3)/pET-28a-YFP, *E. coli* BL21 (DE3)/pET-28a-YFPt-PCDS and *E. coli* BL21 (DE3)/pET-28a-YFP-PCDS refer to ultrasound-broken cells after centrifugation collection of 100ml IPTG induced expression. The amounts of purified His_6_-YFP, His_6_-YFP-PCDS, and His_6_-YFPt-PCDS were detected directly after purification. Protein concentrations were determined with the common Bradford assay (Bradford, 1976).

### Molecular Weight Determination

Molecular weight of the purified recombinant His_6_-PcaHG98e04-PCDS was determined by sodium dodecyl sulfate-polyacrylamide gel electrophoresis (SDS-PAGE) and liquid chromatograph high-resolution accurate mass spectrometry (LC–MS). LC–MS data were obtained using a Thermo Scientific™ Q Exactive™ mass spectrometer (Thermo Fisher Scientific, Waltham, MA, United States). The recombinant His_6_-PcaHG98e04-PCDS was purified by SDS-PAGE. The His_6_-PcaHG98e04-PCDS sample was dissolved in 2% acetonitrile and 0.1% formic acid. The mixture was vortex mixed and centrifuged at 12,000*g* for 15min. The sample was then purified by ultra-filtration, concentrated using Amicon Ultra-10K centrifugal filter device and stored in 0.1% formic acid. MS was performed using a Thermo Scientific Q Exactive mass spectrometer operated in the data-dependent/dynamic exclusion mode. A full resolution setting of 70,000 (full width at half maximum, FWHM) was used for full scan MS. All data were processed using Thermo Scientific™ Pinpoint™ software (Thermo Fisher Scientific, Waltham, MA, United States).

### Enzyme Activity Assays

PCA-3,4-dioxygenase activities were detected by referring to previously described methods (Stanier and Ingraham, 1954; Gross et al., 1956). For the assay of PCA-3,4-dioxygenase activity, the reaction mixture contained 50mM of PB (pH 7.5) and 200μM of cell extracts or purified His_6_-PcaHG98e04-PCDS. All compounds except the substrate were added to the reference cuvette, and the enzyme activity assay was initiated by the addition of 30μg of PCA. The spectra in the range of 220–400nm were recorded every minute. The molar extinction coefficients for PCA at 290nm and 3-carboxy-*cis*, *cis*-muconate at 270nm were 3,890M^−1^ cm^−1^ (Gross et al., 1956) and 6,390M^−1^ cm^−1^ (Iwagami et al., 2000), respectively. One unit of enzyme activity was defined as the protein amount required for the production (or disappearance) of 1μmol of product (or substrate) min^−1^ at 30°C. The protein amounts of *E. coli* BL21 (DE3)/pET-28a-*pcaHG98e01* and *E. coli* BL21 (DE3)/pET-28a-*pcaHG98e04* refer to the soluble protein after the 100ml IPTG induced cell is ultrasonically broken and centrifuged. The amount of purified His6-PcaHG98e04-PCDS was directly detected. All enzyme activity assays were run in triplicate in three independent experiments.

### Fluorescence-Activated Cell Sorting and Fluorescent Spectral Analysis

The BL21 (DE3)/pET-28a(+) was used as a nonfluorescence control. The culture broths of BL21 (DE3)/pET-28a(+), BL21 (DE3)/pET-28a-YFP, BL21 (DE3)/pET-28a-YFP-PCDS, and BL21 (DE3)/pET-28a-YFPt-PCDS grown under the same conditions according to that of YFP expression. Then the cell were pelleted, washed twice with 50mM of Tris-HCl buffer (pH 7.4), resuspended in Tris-HCl buffer, and transferred to a 2-ml round bottom centrifuge tube, respectively. The cell concentration was diluted to 5×10^4^–5×10^5^ and counted. Fluorescence-activated cell sorting (FACS) analysis was carried on a Cytoflex S flow cytometer (Beckman Coulter, IN, United States). The laser beam for cells was 488-nm, and a 529±14-nm FL1 band pass filter used to filter the emission signal. The light signals from the cells are converted to voltage values and the minimum voltage value (400V) was used, with a threshold of 9,200. FACS analysis was run in triplicate in three independent experiments. Data were analyzed using FlowJo v10.0.4 (Tree Star Inc., Ashland, United States) software.

Purified recombinant His_6_-YFP, His_6_-YFP-PCDS, and His_6_-YFPt-PCDS were diluted to 0.1mg/ml with 50mM of Tris-HCl buffer (300mM of NaCl, pH 7.4) and monitored by a Fluorescence Spectrophotometer (F96PRO, Shanghai Kingdak Scientific Instrument, Shanghai, China). The emission and excitation wavelengths of the recombinant protein were 400 and 700nm, respectively. Fluorescent spectral analysis was run in triplicate in three independent experiments.

### Statistical Analysis

The statistical analysis used in this study was performed by SPSS version 23.0 software (IBM SPSS Inc., New York, NY, United States). One-way analysis of variance (ANOVA) was used to calculate the values of *p* for PCA-3,4-dioxygenase activity analyses. Paired-sample tests were used to calculate probability (*p*) values for the expression of YFP. The values of *p* of 0.05 and 0.01 were considered as statistically significant and highly statistically significant, respectively.

## Results

### *pcaHG98* Gene Cloning and Expression

*pcaHG98*, encoded a putative protocatechuate 3,4-dioxygenase, was cloned into pET-28a (+). The *pcaHG98* gene was amplified by PCR with primers pcahg9801 and pcahg9802 (Figure 1) using strain NCIMB 9866 gDNA or bacteria lysate as a template. But the gene could not be amplified into PCR products using either *Taq* DNA polymerase or *Pfu* DNA polymerase. In order to determine this sequence on the genome of strain NCIMB 9866, two primers, pcahg98S01 and pcahg98S02 (Figure 1), were designed for sequencing analysis, and the results show that the sequence was consistent with previous reports (Chen et al., 2014). Then, two primers, pcahg9803 and pcahg9804 were redesigned (Figure 1) successfully cloned the *pcaHG98* gene into pET-28a (+) to generate pET-28a-*pcaHG98e01*.

**Figure 2:**
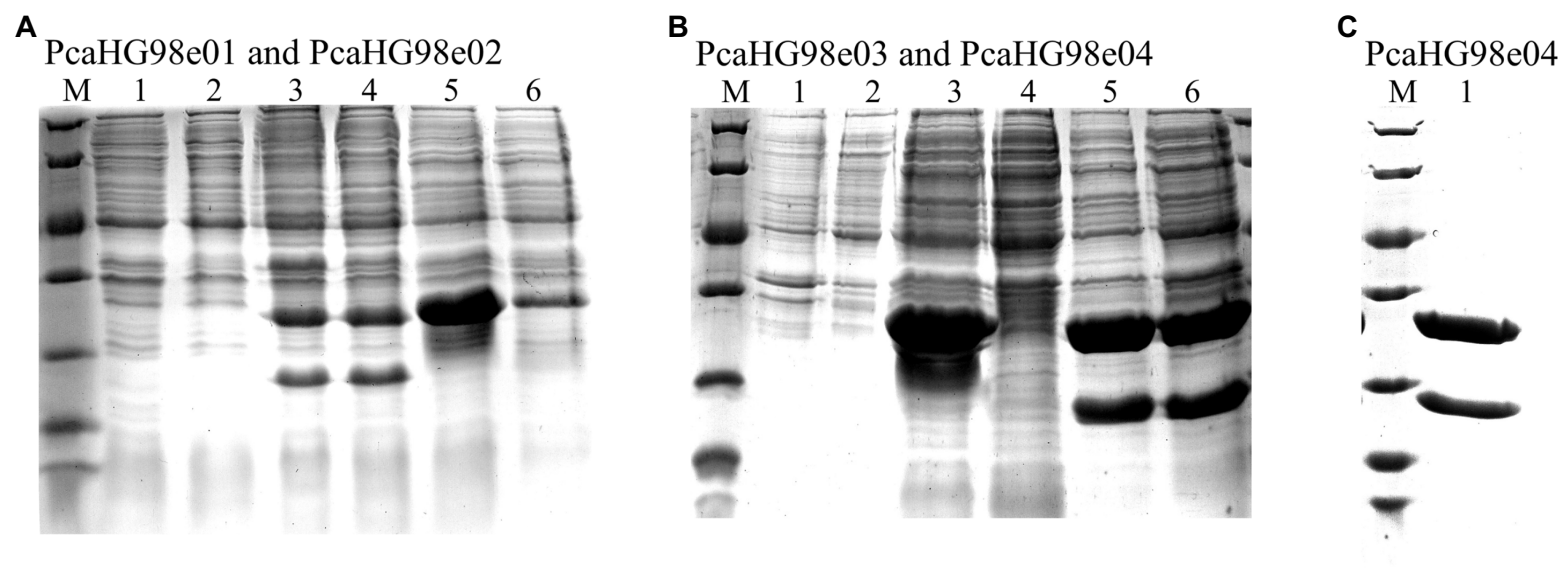
Determination of the expression of recombinant PcaHG in *E*. *coli* BL21 (DE3) by SDS-PAGE. Total cell extract and soluble cell extract from induced *E*. *coli* BL21 (DE3). Lane M, protein molecular mass standards (Unstained Protein Molecular Weight Marker, No. 26610, Thermo Fisher Scientific, Waltham, MA, United States). **(A)** lane 1, total BL21 (DE3)/pET-28a (+), lane 2, soluble BL21 (DE3)/pET-28a (+), lane 3, total BL21 (DE3)/pET-28a-*pcaHG98e01*, lane 4, soluble BL21 (DE3)/pET-28a-*pcaHG98e01*, lane 5, total BL21 (DE3)/pET-28a-*pcaHG98e02*, lane 6, soluble BL21 (DE3)/pET-28a-*pcaHG98e02*. **(B)** lane 1, total BL21 (DE3)/pET-28a (+), lane 2, soluble BL21 (DE3)/pET-28a (+), lane 3, total BL21 (DE3)/pET-28a -*pcaHG98e03*, lane 4, soluble BL21 (DE3)/pET-28a-*pcaHG98e03*, lane 5, total BL21 (DE3)/pET-28a-*pcaHG98e04*, lane 6, soluble BL21 (DE3)/pET-28a-*pcaHG98e04*. **(C)** lane 1, soluble BL21 (DE3)/pET-28a-*pcaHG98e04*; lane 2. Purification of His_6_-PcaHG98e04-PCDS protein from BL21 (DE3)/pET-28a-*pcaHG98e04*.

pET-28a-*pcaHG98e01*, which contained the amplified *pcaHG98* gene from strain NCIMB 9866 gDNA, was expressed in *E. coli* BL21(DE3). The molecular masses calculated from the nucleotide sequence of PcaG and PcaH were 22.7 and 26.3kDa, respectively, which corresponded to the proteins observed by SDS-PAGE (Figure 2A). The PcaHG9801 protein, however, did not bind to Ni^2+^-nitrilotriacetic acid agarose for chromatography. We obtained the same results three separate times. This may have been because the *pcaHG98* gene included 54bp of upstream sequence and 45bp of downstream sequence in pET-28a-*pcaHG98e01*. The start and stop codons of the *pcaHG98* gene still retained with it in this recombinant. Therefore, a His-tag is not fused to the *pcaHG98* gene due to the presence of its start and stop codons.

The *pcaHG98* gene in vector pET-28a-*pcaHG98e02* showed no effective expression, and most of its proteins were insoluble, of which the α-subunit PcaG was not determined by SDS-PAGE (Figure 2A). The *pcaHG98* gene was amplified from pET-28a-*pcaHG98e01* to produce pET-28a-*pcaHG98e03*, in which the *pcaHG98* gene is expressed in the form of inclusion body (Figure 2B). However, the *pcaHG98* gene in pET-28a-*PcaHG98e04* was expressed in the form of soluble proteins, which was amplified using primers pcahg9801 and pcahg9804 (Figure 2B).

### Purification and Biochemical Properties of PcaHG98e04

The PcaHG98e04 protein was constructed using the primers pcahg9801 and pcahg9804, and the primer pcahg9801 (which removed the start codon ATG) starts with the *pcaH* gene 5′-end, with the lead pcahg9804 on the outside of the *pcaG* gene (containing 45 bases: GACATCGCCGGGGCGCGCGCGCG). Compared with the insoluble PcaHG98e02, these 45 bases translated into polypeptides make the His_6_-PcaHG98e04-PCDS protein soluble, the reasons for which are further explored below (Figure 2B).

**Figure 3:**
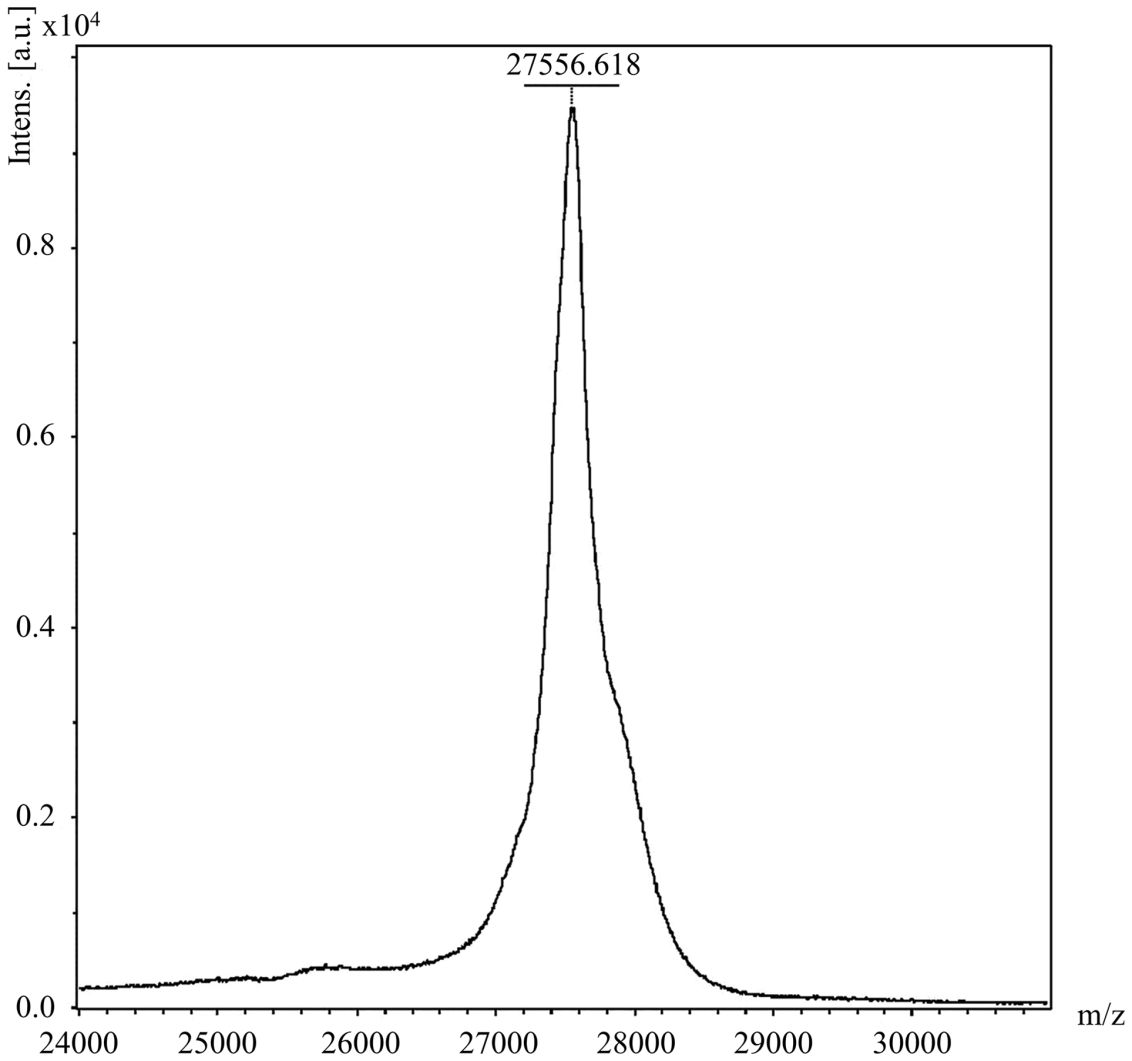
Determination of molecular weight of His_6_-PcaHG98e04 by LC–MS. The mass spectrum of the 27556.618Da His_6_-PcaHG98e04-PCDS protein detected using the Full MS Scan range from 500 to 5,000m/z.

His_6_-PcaHG98e04-PCDS, purified using Ni^2+^-nitrilotriacetic acid agarose chromatography, was then determined using LC–MS. The molecular weight of the protein detected was 27.6kDa (Figure 3), which contained 22.7kDa encoded by the *pcaG* gene and 3.3kDa encoded by the 45-bp (the underlined DNA sequence) and the other end sequences (GACATCGCCGGGGCAGCTGCTCCGGCACGAGTACGAAACGCGTCT CAAGCTTGCGGCCGCACTCGAGCACCACCACCACCACCACTGAGATCCGGCTGCTAA). These results indicated that the expressed protein PcaG contained the PCDS tag.

### His_6_-PcaHG98e04-PCDS Catalyzes the Oxidization of PCA

**Figure 4:**
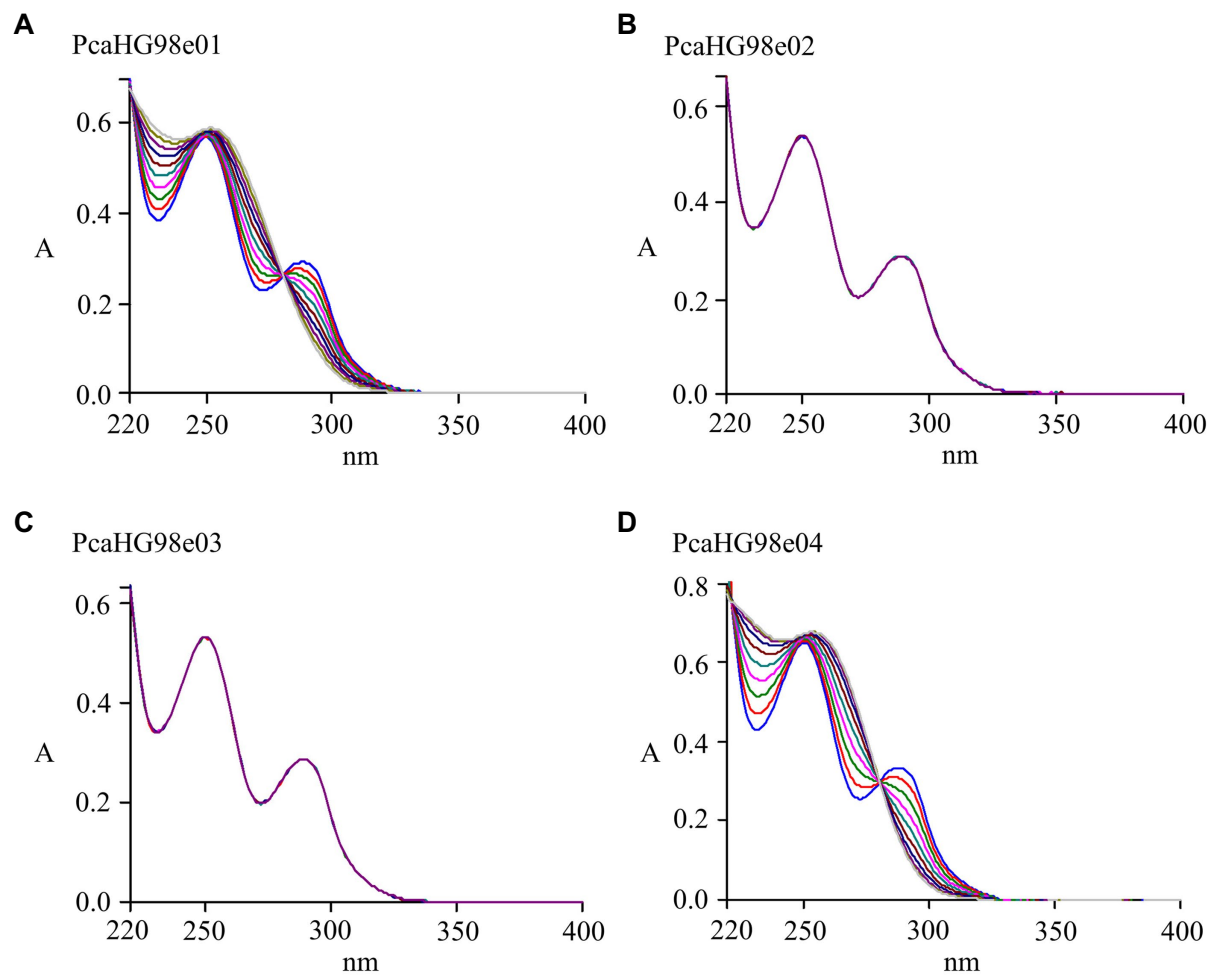
Determination of *pcaHG98*-encoded PCA 3,4 dioxygenase activity. Spectrophotometric changes during oxidation of PCA by *pcaHG98*-encoded PCA 3,4 dioxygenase. The assay of PCA catalyzed by cell extracts from *E*. *coli* BL21 (DE3) carrying pET-28a-*pcaHG98e01* **(A)** or pET-28a-*pcaHG98e04* **(D)**. There was no enzyme activity in the cell extracts from BL21 (DE3) carrying pET-28a-*pcaHG98e02* **(B)** or pET-28a-*pcaHG98e03* **(C)**.

Protocatechuate 3,4-dioxygenase catalyzes the cleavage of PCA into β-carboxy-*cis*, *cis*-muconate (Harwood and Parales, 1996). A series of DNA fragments of different lengths containing the complete reading frame of the *pcaHG98* gene was ligated into the expression vector pET-28a (+), to make the plasmids pET-28a-*pcaHG98e01*, pET-28a-*pcaHG98e02*, pET-28a-*pcaHG98e03*, and pET-28a-*PcaHG98e04*. These recombinant plasmids were then individually expressed in *E. coli* BL21 (DE3). After induction with IPTG, *E. coli* BL21 (DE3)/pET-28a-*pcaHG98e01* and *E. coli* BL21 (DE3)/pET-28a-*pcaHG98e04* cell extracts were found to contain PCA 3,4-dioxygenase with specific activities of 74.7±5.31 and 72.8±4.41U/mg against PCA as a substrate, respectively. No activity was detectable in *E. coli* BL21 (DE3)/pET-28a-*pcaHG98e02* and *E. coli* BL21 (DE3)/pET-28a-*pcaHG98e03*, where the expression vector contained no soluble PcaHG98 protein. Purified His_6_-PcaHG98e04-PCDS exhibited 205.63±14.23U/mg activity against PCA as a substrate, which was not affected by the PCDS tag. Figure 4 shows the rapid transformation of PCA (*λ*_max_=290nm) to 3-carboxy-*cis*, *cis*-muconate (*λ*_max_=270nm) in the cell extract, as described by Harwood and Parales (1996).

### Biochemical Properties of Recombinant His6-YFP, His6-YFP-PCDS, and His6-YFPt-PCDS

**Figure 5:**
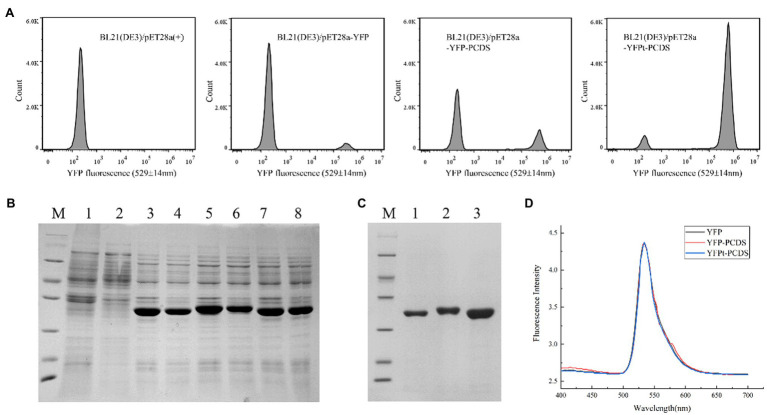
Biochemical properties of recombinant His_6_-YFP, His_6_-YFP-PCDS, and His_6_-YFPt-PCDS. **(A)** Expression levels of BL21 (DE3)/pET-28a(+), BL21 (DE3)/pET-28a-YFP, BL21 (DE3)/pET-28a-YFP-PCDS and BL21 (DE3)/pET-28a-YFPt-PCDS were analyzed by FACS. **(B)** Total cell extract and soluble cell extract from induced *E*. *coli* BL21(DE3). Lane M, protein molecular mass standards (Unstained Protein Molecular Weight Marker, No. 26610, Thermo Fisher Scientific, Waltham, MA, United States); lane 1, total BL21 (DE3)/pET-28a (+); lane 2, soluble BL21 (DE3)/pET-28a (+); lane 3, total BL21 (DE3)/pET-28a-YFP; lane 4, soluble BL21 (DE3)/pET-28a-YFP; lane 5, total BL21 (DE3)/pET-28a-YFP-PCDS; lane 6, soluble BL21 (DE3) /pET-28a-YFP-PCDS; lane 5, total BL21 (DE3)/pET-28a-YFP-PCDS; lane 6, soluble BL21 (DE3)/pET-28a-YFP*-*PCDS; lane 7, total BL21 (DE3)/pET-28a-YFPt*-*PCDS; lane 8, soluble BL21 (DE3)/pET-28a-YFPt-PCDS. **(C)** Purified soluble protein. Lane M, protein molecular mass standards (Unstained Protein Molecular Weight Marker, No. 26610, Thermo Fisher Scientific, Waltham, MA, USA); lane 1, His_6_-YFP; lane 2, His_6_-YFP-PCDS; lane 3, His_6_-YFPt-PCDS. **(D)** Spectroscopic intensity measurements of His_6_-YFP (black line), His_6_-YFP-PCDS (red line), and His_6_-YFPt-PCDS (blue line).

It has been reported that the YFP has relatively low expression in *E. coli* compared to that of codon-optimized YFP (Nguyen et al., 2019). The YFP gene was synthesized according to the sequence of the mammalian expression vector pcDNA3YFP and cloned into the *E. coli* expression vector pET28a (+) in this study. Based on our results, the YFP gene was expressed in our *E. coli* system and had a certain amount of solubility (Figure 5B). The 45-bp PCDS tag enhanced the soluble expression of the YFP gene in *E. coli* after fusing the PCDS tag to the YFP C-terminus (Figure 5B). This YFP-PCDS did not have YFP termination codons, whereas YFPt-PCDS contained YFP termination codons. The PCDS tag enhanced the expression level of YFP in *E. coli* was analyzed by fluorescence-activated cell sorting (FACS). FACS analysis showed that the fluorescence intensity of BL21 (DE3)/pET-28a-YFP-PCDS and BL21 (DE3)/pET-28a-YFPt-PCDS was higher than that of BL21 (DE3)/pET-28a-YFP, and BL21 (DE3)/pET-28a-YFPt-PCDS was the highest (Figure 5A). BL21 (DE3)/pET-28a-YFP showed weaker fluorescence intensity, as did the control BL21 (DE3)/pET-28a(+). The total protein expression of YFP-PCDS and YFPt-PCDS was significantly amplified up to 1.6-fold and 2-fold compared to that of YFP alone, respectively. Accordingly, His_6_-YFP-PCDS and His_6_-YFPt-PCDS had 1.6-fold and 3-fold higher soluble expression yield, respectively, than that of His_6_-YFP under the same expression and purification conditions (Figure 5C). All protein amounts were determined three times in three independent experiments. His_6_-YFP, His_6_-YFP-PCDS, and His_6_-YFPt-PCDS also showed consistent fluorescence emission spectra, with a peak at 530nm across the scanning range from 400 to 700nm (Figure 5D).

## Discussion

The β-ketoadipate pathway is preceded by the preliminary conversion of a broad range of organic compounds into one of two aromatic rings, catechol or PCA (Harwood and Parales, 1996; Wells and Ragauskas, 2012). Protocatechuate 3,4-dioxygenase catalyzes the cleavage of PCA into β-carboxy-*cis*, *cis*-muconate (Harwood and Parales, 1996). *pcaHG98* encodes a putative protocatechuate 3,4-dioxygenase in NCIMB 9866. This gene, unfortunately, cannot be directly amplified by PCR with primers pcahg9801 and pcahg9802 using strain NCIMB 9866 gDNA or bacteria lysate as a template using either the *Taq* or *Pfu* DNA polymerase. Sequencing analysis results show that the sequence of the *pcaHG98* gene was consistent with genome sequencing data. Then, two primers, pcahg9803 and pcahg9804 were redesigned (Figure 1), and the *pcaHG98* gene was successfully cloned into pET-28a (+) to generate pET-28a-*pcaHG98e01*. The recombinantly expressed PcaHG98e01 (Figure 2A) had protocatechuate 3,4-dioxygenase activity (Figure 4A). These results show that the 54bp and 45bp of the *pcaHG98e01* gene (Figure 1) contain the sequences needed for its solubility expression, which were also beneficial for gene amplification. In order to determine the role of these two sequences, we constructed pET-28a-*pcaHG98e03*, which contained 54bp on the N terminus, and pET-28a-*pcaHG98e04*, which contained 45bp on the C terminus (Figure 1). The PcaHG98e03 protein was insoluble (Figure 2B), and the PCA 3,4-dioxygenase activity was not detectable in *E. coli* BL21 (DE3)/pET-28a-*pcaHG98e03* (Figure 4C), while the PcaHG98e04 protein was soluble (Figure 2B), and *E. coli* BL21 (DE3)/pET-28a-*pcaHG98e04* cell extracts were found to exhibit activity, with a specific activity of 72.8±4.41U/mg against PCA as a substrate. Therefore, the 45bp on the C terminus of the *pcaHG98e04* gene was beneficial for its soluble protein expression, which we named as the PCDS tag.

**Figure 6:**
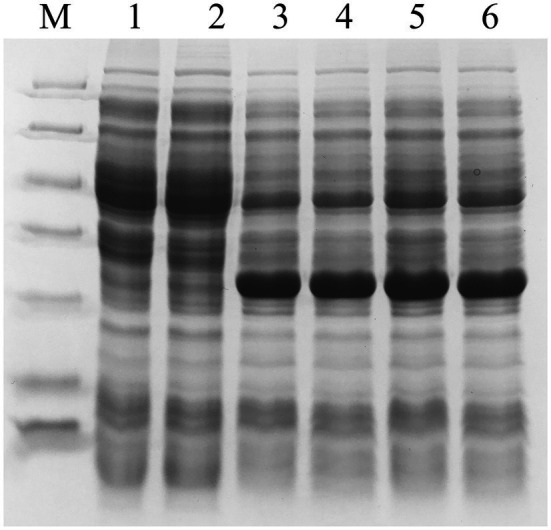
Determination of the expression of recombinant eGFP in *E. coli* BL21 (DE3) by SDS-PAGE. The total cell extract and soluble cell extract from induced *E*. *coli* BL21 (DE3). Lane M, protein molecular mass standards (Unstained Protein Molecular Weight Marker, No. 26610, Thermo Fisher Scientific, Waltham, MA, United States); lane 1, total BL21 (DE3)/pET-28a (+), lane 2, soluble BL21 (DE3)/pET-28a (+), lane 3, total BL21 (DE3)/pET-28a-eGFP, lane 4, soluble BL21 (DE3)/pET-28a-eGFP, lane 5, total BL21 (DE3)/pET-28a-eGFP-PCDS, lane 6, soluble BL21 (DE3)/pET-28a-eGFP-PCDS.

In order to analyze the solubility impact of the PCDS tag, we selected YFP, with its pI of 5.910 and its relatively low level of expression in *E. coli* according to the literature (Nguyen et al., 2019). Based on our results, the YFP gene was expressed in the *E. coli* system with a certain amount of solubility (Figure 5B). FACS analysis showed that the fluorescence intensity of YFP-PCDS and YFPt-PCDS in *E. coli* BL21(DE3) was higher than that of YFP, and YFPt-PCDS was the highest (Figure 5A). The PCDS tag could enhance the soluble expression of the YFP gene by fusing the PCDS tag to the YFP C-terminus in *E. coli* (Figure 5B). The total protein expression of YFP-PCDS and YFPt-PCDS without and with a termination codon was significantly amplified up to 1.6-fold and 2-fold compared to that of YFP alone, respectively. Accordingly, His_6_-YFP-PCDS and His_6_-YFPt-PCDS had 1.6-fold and 3-fold higher soluble expression yield, respectively, than that of His_6_-YFP under the same conditions. They also showed a consistent fluorescence emission spectra, with a peak at 530nm over a scanning range from 400 to 700nm (Figure 5D). This result also showed that the presence of the stop codon also affected the improvement in solubility induced by the PCDS tag, which was located at a similar position as the *pcaHG98* gene. In addition, the enhanced green fluorescent protein (eGFP) gene is also less expressed in the *E. coli* expression system and has been used for fusion tagging (Marblestone et al., 2006). Herein, the eGFP gene originated from the mammalian expression vector pEGFP-N1 was cloned into pET-28a (+) to produce pET-28a-eGFP-PCDS. Induced expression results (Figure 6) show that the recombinant eGFP (a pI of 5.731) expressed in *E. coli* BL21 (DE3)/pET-28a-eGFP-PCDS was basically all soluble, which did not show the solubility effect of this 45-bp PCDS tag.

The acidity of fusion tags can significantly improve the solubility of fusion proteins; so, acidic fusion tags that are negatively charged at physiological pHs have been widely screened and are widely used (Ki and Pack, 2020). However, the 15 amino acid PCDS tag in this study was an alkaline fusion tag with a pI value of 9.849 and six basic amino acid arginines. The pI value of PcaH and PcaG was 9.599 and 4.378, respectively. A PCDS tag located at the C-terminus of PcaG may promote PcaHG soluble expression by influencing the PcaG protein. The PCDS tag found in this study may be more suitable for insoluble expression due to protein acidity, which requires further analysis. In addition, the 45-bp sequence outside the end of the *pcaHG98* gene affects its PCR cloning from the genome, but the reason is confusing and needs further analysis. A protein has its lowest solubility when the ambient pH is equal to its pI, at which point it shows a zero net charge. Therefore, optimizing the net charge to a positive or negative charge improves the solubility of a protein (Lawrence et al., 2007). Depending on the pI value of the target protein, the use of fusion tags to induce a rejection of static interaction between proteins can provide sufficient time for the correct folding of proteins, thus preventing protein aggregation (Zhang et al., 2004; Kato et al., 2007; Paraskevopoulou and Falcone, 2018). The pI values of PcaH and PcaG were 9.599 and 4.378, respectively. The PCDS tag having a high pI value of 9.849 may promote PcaHG soluble expression by influencing the static interaction between them. In this respect, the PCDS tag is similar to the protein fusion tag CBD. CBD also has a high pI value (Hopp et al., 1988; Marblestone et al., 2006) that improves the soluble expression of heterogeneous proteins in *E. coli* (Murashima et al., 2003). Most peptide fusion tags are located at the N-terminus of an expression protein, which promotes the correct folding of a protein to enable its solubility expression by affecting transcription initiation (Seo et al., 2013; Nguyen et al., 2019). In contrast to this, some studies have shown that poly-lysine or poly-arginine tags fused to the C-terminus of a target protein are more likely to enhance its solubility than a tag fused to the N-terminus (Park et al., 2003; Hage et al., 2015; Islam et al., 2015; Nautiyal and Kuroda, 2018). A PCDS tag located at the C-terminus of PcaG may promote PcaHG soluble expression by influencing the static interaction between them.

In fact, the soluble expression of recombinant proteins is controlled by many factors, including the host organism, the types of expression vector used, codon bias, culture conditions, transcription initiation, mRNA stability, and protein toxicity (Rosano and Ceccarelli, 2014). An inclusion body is a common hurdle for the heterologous expression of recombinant proteins, which is usually solved by various solubility-enhancing tags, including protein fusion tags and peptide tags (Paraskevopoulou and Falcone, 2018). These peptide tags have been developed and applied to enhance heterologous protein expression, including FLAG-tags (Einhauer and Jungbauer, 2001), Arg-tags (Sassenfeld and Brewer, 1984; Terpe, 2003), Fh8 and H tags (Costa et al., 2013), and NT11-tags (Table 3; Terpe, 2003; Nguyen et al., 2019; Ki and Pack, 2020). The advantage of these peptide tags for heterologous expression is that their amino acid sequences usually only have 15 residues or less and therefore do not affect the structure or activity of heterologous proteins (Kato et al., 2007; Paraskevopoulou and Falcone, 2018; Nguyen et al., 2019; Ki and Pack, 2020). Thus, it may not be necessary to remove these peptide tags for further applications of proteins, in contrast to the case of the larger protein fusion tags. In addition, protein and peptide fusion tags are one of the most important methods to increase target protein expression, including expression efficiency and solubility. Therefore, understanding the physicochemical properties of proteins, including their pI value, net charge, and GRAVY values, can help to select and design appropriate fusion tags. Although many protein labels and peptide tags have been reported to promote protein soluble expression, there are still other proteins that cannot achieve heterologous soluble expression, which requires research and development that focused on more and more extensive technologies and fusion tags.

**Table 3:** Peptide tags to improve heterologous protein expression in *Escherichia coli*.

Tag	Sequence	Number of amino acids	pI	Net charge at pH 7	Size (Da)	GRAVY	Tagged position (terminus)	References
NT11	VSEPHDYNYEK	11	4.31	−2	1,380	−1.991	N-	Nguyen et al., 2019
SAP (S1)	(AEAEAKAK)2	16	7.11	0	1,614	−0.950	N-	Zhao et al., 2019
S1nv10	(ANANARAR)2	16	12.96	+ 4	1,667	−1.100	N-	Zhao et al., 2019
S1hv1	(IEIEIKIK)2	16	7.08	0	1951	0.400	N-	Zhao et al., 2019
S1hv3	(VEVEVKVK)2	16	7.08	0	1839	0.250	N-	Zhao et al., 2019
S1V1	(AEAEAHAH)2	16	5.06	−4	1,651	−0.775	N-	Zhao et al., 2018
DDTs	GRGRSRA	7	12.79	+ 3	758	−1.900	N-	Hirose et al., 2011
	KRARAAA	7	12.49	+ 3	742	−0.814	N-	Hirose et al., 2011
S Tag	KETAAAKFERQHMDS	15	7.54	0	1754	−1.393	N-	Kim and Raines, 1993
T7B9	EEASVTSTEETLTPAQEAAE TEANKARKEAELEAETAEQ	39	3.6	−9	4,261	−1.060	N-/C-	Zhang et al., 2004
6×Lys	KKKKKK	6	11.33	+7	914	−3.900	C-	Hage et al., 2015
GFIL8	GFILGFIL	8	5.58	0	879	2.675	C-	Wang et al., 2015
L6KD	LLLLLLKD	8	6.71	0	940	1.925	C-	Zhou et al., 2012
ELK16	LELELKLKLELELKLK	16	7.08	0	1951	0.050	C-	Wu et al., 2011
A18	EWLKAFYEKVLEKLKELF	18	6.95	0	2,312	−0.278	C-	Xing et al., 2011
SEP	GRRRGRRRGRRR	12	13.403	9	1,595	−3.475	C-	Nautiyal and Kuroda, 2018
PCDS	DIAGAAAPARVRNAS	15	9.849	1	1,440	0.047	C-	This study

## Data Availability Statement

The raw data supporting the conclusions of this article will be made available by the authors, without undue reservation.

## Author Contributions

LZ and SL constructed the plasmids, cultured bacteria, and analyzed the data. NL and S-LR analyzed the data. H-JC designed and supervised the study. LZ, SL, JC, JW, DY, and H-JC wrote and revised the manuscript. All authors contributed to the article and approved the submitted version.

## Funding

This work was supported by grants from the National Natural Science Foundation of China (NSFC; grant numbers 31770119, 31400068, and 32070098). The funding organization did not influence the design of the experiment or analysis, and interpretation of data, or preparing the manuscript.

## Conflict of Interest

The authors declare that the research was conducted in the absence of any commercial or financial relationships that could be construed as a potential conflict of interest.

## Publisher’s Note

All claims expressed in this article are solely those of the authors and do not necessarily represent those of their affiliated organizations, or those of the publisher, the editors and the reviewers. Any product that may be evaluated in this article, or claim that may be made by its manufacturer, is not guaranteed or endorsed by the publisher.
